# Construction of PR39 recombinant AAV under control of the HRE promoter and the effect of recombinant AAV on gene therapy of ischemic heart disease

**DOI:** 10.3892/etm.2012.674

**Published:** 2012-08-20

**Authors:** LIJUN SUN, YUEWEN HAO, XIAOWEI NIE, XUEXIN ZHANG, GUANGXIAO YANG, QUANYING WANG

**Affiliations:** 1 Department of Radiology, Xijing Hospital, The Fourth Military Medical University, Xi’an, Shaanxi 710032;; 2 Xi’an Huaguang Bioengineering Limited Company, Xi’an, Shaanxi 710025, P.R. China

**Keywords:** hypoxia-responsive enhancer, hypoxia induction, gene therapy, myocardial infarction

## Abstract

The objective of this study was to investigate the effect of the PR39 recombinant adeno-associated virus (AAV) controlled by the hypoxia-responsive element (HRE) on gene therapy of ischemic heart disease. The minimal HRE was artificially synthesized and the AAV vector controlled by HRE was introduced with NT4-TAT-His-PR39 to investigate the expression of AAV-PR39 in hypoxic vascular endothelial cells (VEC) of human umbilical vein (CRL-1730 cell line) and the angiogenesis-promoting effect in pigs with acute myocardial infraction (AMI). The minimal HRE/CMV was designed and artificially synthesized using the PCR method and cloned with the T vector cloning method. The pSS-HRE-CMV-NT4-6His-PR39-PolyA-AAV plasmid was constructed. Using the calcium phosphate precipitation method, HEK-293 cells were co-transfected with three plasmids to produce the recombinant virus. An equal volume of pSS-HRE-CMV-NT4-6His-PR39-PolyAAAV and enterovirus (EV, blank virus) was transfected into CRL-1730 cell lines, respectively. The immunohistochemical method was used to assay the expression of 6xHis in CRL-1730 cell lines and the expression of PR39 under hypoxia. Eighteen AMI miniature pigs were randomized into the experimental group (HRE-AAV-PR39 group), control group 1 (physical saline group) and control group 2 (EV group). The area of ischemia was assessed with conventional MRI and myocardium perfusion MRI. Pigs were sacrificed at preset time-points to obtain samples of ischemic myocardium. Morphological and pathological data were collected. According to data in the literature and databases, the minimal HRE was designed and synthesized with the PCR method. A large number of HREs were connected to modified pSSHGAAV (pSSV9int-/*Xba*I) vector followed by insertion of the NT4-6His-PR39 gene segment and, thus, the recombinant plasmid pSS-HRE-CMV-NT4-6His-PR39-PolyA-AAV was successfully constructed. The expression of 6xHis in CRL-1730 cells under the regulation of HRE was assayed using the immunohistochemical method and results showed that the expression was positive in the experimental group. Myocardium perfusion MRI displayed that the infracted area significantly decreased under the action of pSS-HRE-CMV-NT4-PR39-PolyA-AAV. The artificial minimal HRE in CRL-1730 cells effectively and rapidly regulates the expression of the downstream gene NT4-TAT-His-PR39 of the CMV promoter. Recombinant pSS-HRE-CMV-NT4-PR39-Poly-AAAV promotes neoangiogenesis in the ischemic area, reduces the area of infarction and improves heart function.

## Introduction

The hypoxia-responsive element (HRE) is the minimal indirect *cis*-regulatory element transactivated by hypoxia-inducible factor (HIF). Data from over 70 genes suggest that endogenous HREs are composite regulatory elements comprising the conserved HIF-binding site (HBS) with an A/GCGTG core sequence and a highly variable flanking sequence. A single HBS is essential but not sufficient for activation by hypoxia. Binding sites of transcription factors provided by the flanking sequence are not necessary for hypoxia regulation but are required to amplify the hypoxic response or make the HRE tissue-specific (1). In contrast to regulation of the HIF pathway, our knowledge of the fundamental structural features of HREs is quite limited. In this study, we firstly synthesized the minimal HRE and then introduced NT4-TAT-His-PR39 to HRE-regulated adeno-associated virus (AAV) to investigate the expression in hypoxic vascular endothelial cells (VEC) of human umbilical vein (CRL-1730 cell lines) and the angiogenesis-promoting effect on myocardial cells in pigs with acute myocardial infarction (AMI).

## Materials and methods

### 

#### Cell line, plasmid, tool enzyme, cell and cloning vector

*Escherichia coli* TOPIO, T/T AT-His vector, pBV220/NT4 vector, AAV vector (pSSHG-CMV), helper virus pAAV/Ad, helper packaging plasmid PFG140 and biological enzyme were purchased from the Xi’an Sino-American Biotechnology Co., Ltd. Hypoxic (1% O_2_) incubator was provided by the Department of Pathology, School of Basic Medical Sciences, Fourth Military Medical University. CRL-1730 cells were provided by the Department of Pharmacology, Xian Jiaotong University. CoCl_2_ was provided by the Department of Molecular Genetics, Fourth Military Medical University. Recombinant virus pSS-HRE-CMV-NT4-TAT-6His-PR39-PolyA-AAV was packed. Experimental miniature pigs were purchased from the Animal Center, Fourth Military Medical University (Chengdu Dossy Biological Technology Co., Ltd.). Pentobarbital was purchased from the Reagent Center, Fourth Military Medical University. Suminaxin injection was manufactured by the Jilin Huamu Animal Health Product Co., Ltd. The experimental procedures in the present study were approved by the local ethics committee and the animals were treated according to guidelines set by our University’s Animal Laboratory (The Fourth Military Medical University, Shaanxi, China).

### Synthesis and cloning of infusion gene HRE-CMV-MCS-PolyA

*Design and synthesis of primers.* According to data in the literature and databases, the minimal HRE sequence was CTGCACGTA CTGCACGTA CTGCACGTA CTGCACGTA and 35 bp sequences spacing in the TA Box. Based on nucleotide sequences in GenBank, *Xba*I-recognition sequence TCTAGA (cohesive end) was added to the 5′ and 3′ ends and the protective base G was introduced to the 5′ end of forward and reverse primers. Using the DNAsis software, forward and reverse primers were designed as follows and were synthesized by the Shanghai Generay Biotech Co., Ltd.: F1, ‘-g 5′-*TCTAGA* CTGCACGTA CTGCACGTA CTGCACGTA CTGCACGTA atcaattacg gggtcattag-3′; ‘-g 5′-*TCTAGA* acgcgttaag atacattgat gag-3′. The complete pGEM-T-HRE-CMV-MCS-PolyA was obtained by PCR amplification and this sequence was completely consistent with that of gene expression box designed, as proved by DNAsis software.

*Construction of pSS-HRE-CMV-MCS-PolyA-AAV vector.* pSS-HRE-CMV was digested with both E. co 721 and *Bam*HI. The digested HRE-CMV segment was connected with the human pBV220/NT4-TAT-His-PR39 vector with the method aforementioned. The recombinant T/TAT-His-PR39 was harvested and digested by both *Nae*I and *Bam*HI. The digested segment was connected with the human pBV220/NT4 vector. The recombinant pBV220/NT4-TAT-His-PR39 was harvested and sent to Sangon Biotech (Shanghai) Co., let for gene sequencing.

*Package of recombinant virus*. Using the calcium phosphate precipitation method, HEK-293 cells were co-transfected with plasmid pssHG-9H-CMV-NT4-PR39 helper packaging plasmid pAAV/Ad with cloning Rep and Cap genes, and adenoviral plasmid PFG140 to produce the recombinant AAV-9H-CMV-NT4-PR39.

### Recombinant AAV under HRE promoter control for myocardium of ischemic heart disease (IHD) pigs

*Construction of myocardial infarction in miniature pigs*. Eighteen miniature pigs were randomized into the experimental group (HRE-AAV-PR39 group, n=8), control group 1 (physical saline group, n=4) and 2 (EV group, n=4). Every pig was fasted for 8 h and then injected intramuscularly with 2 ml of Suxinmian injection and 18 ml of 5% pentobarbital (1 ml/kg). Under electrocardiographic (ECG) monitoring, left ventriculography was performed by introducing the guide wire and 6F arterial sheath; subsequently, 6F Amplatz guide catheter was exchanged to conduct coronary angiography. The occluded site of the left anterior descending artery (LAD) was identified. Next, a PTCA balloon catheter was introduced into the distal LAD artery with the aid of a microcatheter and microguide wire. The balloon (2.0 or 2.5) was placed 0.5–1.0 cm below the first diagonal branch of LAD and inflated with 4–6 times of atmospheric pressure to occlude LAD for 90 min. If LAD was completely occluded, the AMI model was successfully constructed. One milliliter of AAV/HRE-PR39 or 1 ml of physical saline/control virus was injected through the microcatheter. After that, the catheter and sheath were removed. Magnetic resonance imaging (MRI) was performed preoperatively and 24 h, 1, 2 and 3 weeks postoperatively.

*MRI scanning post AMI.* In this study, a magnetom triotim 3.0-T magnetic resonance scanner (Siemens, German) was used to perform MRI scanning under both the conventional MR sequence and a novel sequence sensitive to myocardium edema (T_2_-weighted TrueFISP imaging of myocardium). Additionally, myocardium delayed perfusion scanning was also performed under the TrueFISP-PSIR sequence. Changes in MR images of MI area over time were observed. Bright-blood sequence: scanning was performed using the novel sequence sensitive to myocardium edema (T_2_-weighted TrueFISP imaging of myocardium). Scanning parameters were as follows: TR/TE, 281.95/1.09 msec; section thickness, 6 mm; gap, 0; matrix, 256x256; FOV, 360 mm; RFOV, 75%; acquisition window, 715 msec; 2 trigger pulse, 2; trigger delay, 433 msec and Flip, 90°C. For the TrueFISP-PSIR sequence to performed myocardium delayed perfusion scanning, 2D TrueFISP-PSIR sequence was used. The contrast agent was injected at a rate of 0.1 mmol/kg. Scanning was started 10 min later. Scanning parameters were as follows: TR/TE, 82.24/1.09 msec; section thickness, 6 mm; gap, 0; matrix, 256x256; FOV, 360 mm; RFOV, 75%; acquisition window, 750 msec; 2 trigger pulse, 2; trigger delay, 300 msec and Flip, 90°C.

#### Molecular assessment after AMI

A pig of every group was sacrificed respectively 1, 2 and 3 weeks postoperatively. The fresh heart was harvested for immunohistochemical examination to measure expression of HIF-1α at the area of ischemia. The myocardium tissue was fixed with paraform, dehydrated with 60–100% alcohol solution, and was embedded in soft, neutral and hard paraffin wax. Sections were made followed by dewaxing in 170–100%. Ischemia myocardium samples of each pig were collected for SABC immunohistochemical staining to observe expression of HIF-1α around the area of infarction and compare expression levels between the experimental and control groups.

## Results

### 

#### Expression of HRE-triggering AAV-PR39 under hypoxia

**Figure 1:**
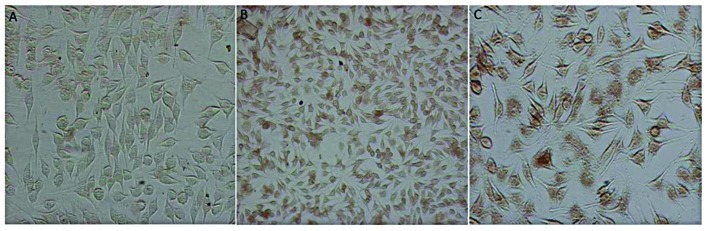
Expression of 6xHis in CRL-1730 cells by immunohistochemical examination. (A) The EV group under normal oxygen (x400); (B) The AAV/HRE-PR39 group under the normal oxygen (x200); (C) The AAV/HRE-PR39 group under hypoxia (x400).

The OD of the 6xHis staining area was calculated with the IPP Image Processing software, which represented the level of PR39 protein. In the AAV/HRE-PR39 group, massive brown area was present in and out of the cytoplasm under hypoxia but only in the cytoplasm under common oxygen; in non-viral cells, a small quantity of brown area was observed in the cytoplasm (a trace of 6xHis background staining was present in the normal cytoplasm). The average OD value for positive 6xHis was 132.3±8.5 in control group 2, 23.3±5.4 in the experimental group and 48.5±7.3 in control group 1 (control group 2 vs. experimental group, P<0.05; control group 1 vs. 2, P<0.05), illustrating that HRE triggers the expression of NT4-TAT-His-PR39 of hypoxic CRL-1730 cells (Fig. 1).

#### Imaging results

Delayed perfusion MR images in different stages of AMI models could significantly distinguish normal and infarcted myocardium. The area of infarction was displayed clearly. Significant differences in myocardium changes over time post AMI were noted between the experimental and control group. The area of infarction in the control group changed slightly over time (no inflammatory edema period was taken into account). In the experimental group, the area of infarction decreased over time and the difference was statistically significant, as measured by the eFilm software. The left ventricular ejection fraction post AMI also differed significantly between the two groups.

#### Pathological results

**Figure 2:**
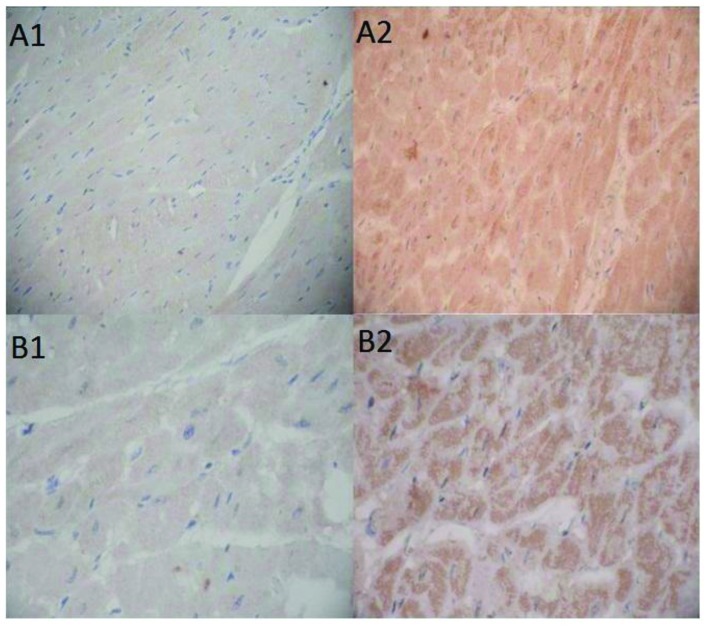
Expression of HIF-1α in myocardial cells by immunohistochemical examination. Brown. HIF-1α protein staining; blue, myocardial nucleus. (A1) The control group; (A2) the experimental group (x200); (B1) the control group; (B2) the experimental group (x400).

Pathological examinations showed that the cardiac apex, the anterior wall of the left ventricle, and the anterior septal were infarcted. The infarcted myocardium was crisp and white and significantly decreased over time in the experimental group. The white myocardium was collected for paraffin sectioning. Expression of HIF-1α in myocardial cells was assayed using immunohistochemistry, as described previously. Digital microphotographs showed that the average OD value for positive HIF-1α was 5469±395.7 in the experimental group and 1686±423.2 in the control group (P<0.001), respectively, suggesting that rAAV expressing PR39 could significantly increase the level of HIF-1α proteins in hypoxic myocardial cells (Fig. 2).

## Discussion

The molecular biological mechanism of cell hypoxia inhibiting responsive regulation suggests that intracellular HIF is activated and highly expressed under hypoxia; furthermore, it can bind the expression regulatory element of hypoxic reactive genes, HRE, triggering and enhancing expression of hypoxic reactive proteins and cytokines via *cis*-activation (2–6). Various HRE-controlled expression vectors have been constructed since 1991 when Semenza *et al* (7) discovered HRE. However, HREs differ greatly in the structure, sequence and hypoxic inducible ability with hypoxic responsive genes (9). A single HRE is not enough to induce a hypoxic response and hypoxic-responsive regulatory vectors often require tandem repeat HREs. However, multiple repeats necessarily increase the sequence length of regulatory elements and the number of genetic elements introduced in the human body and cells.

In 2008, Kaluz *et al* (10) analyzed HRE and HBS of multiple hypoxic responsive genes and the structure and sequence of the artificial-synthesized minimal HRE (36 bp) with the monomer, forward/reverse tandem sequences and point mutation method, which effectively regulated hypoxic expression. It was proven that the 36-bp HRE had the optimal distance with the TATA Box of the CMV promoter and that its hypoxic-inducible ability was higher than 4xEPO HRE and comparable with the CMV promoter.

If the CMV promoter of cytomegalovirus was adopted as the promoter of adenovirus, the downstream PR39 gene will continuously express under any environment (hypoxia or normal oxygen), inducing excessive expression of VEGF protein. VEGF will accumulate in the systemic circulation, causing hemangioma (11,12). In this study, the 9HRE segment was added to the upstream of the AAV promoter CMV, which rapidly triggers the expression of PR39 in myocardial cells under hypoxia and timely terminates the expression of PR39 when normal oxygen returns. Thus, some adverse effects associated with continuous high expression of VEGF are prevented and thus, PR39 is more safe and effective in clinical gene therapy.

According to previous literature, the minimal HRE is planned to insert to the site of 35 bp in the upstream of CMV. However, there is no digestion site in the upstream of the CMV promoter (13). Therefore, the synthetic 36 bp HRE and the 35 bp sequence in the upstream of the CMV promoter (TATA Box) are amplified to the HRE-regulated CMV promoter with PCR technique utilizing the plasmid containing the CMV promoter as the template in accordance with the base complementary principle. The complete HRE-CMV-MCS-PolyA gene sequence is 918 bp and the minimal HRE is the four repeats of CTGCACGTA, easily resulting in the palindrome structure and other mismatching modes during PCR synthesis. In our study, the reactant amount and reaction conditions of the PCR reactive system were adjusted multiple times and HRE-CMV-MCS-PolyA was successfully synthesized and verified by gene sequencing.

In this study, AAV was used as the viral vector carrying PR39, which could stably express PR39 in the host cell during a long period. Recovery of AMI is time enduring. Hence, AAV-PR39 is superior to the conventional method for treating AMI and also valuable in the prevention and management of AMI. It is promising for thr gene therapy of IHD.

The HRE-CMV-MCS-PolyA gene segment is a hypoxia-regulatory promoter requiring increasing research for hypoxia-regulation. This promoter has more potent hypoxia-inducible ability and is more minor than current hypoxia-regulatory promoters. It offers a minor promoter with the regulating ability for constructing thr rAAV vector, i.e. it offers a relatively large remaining space to insert the objective gene for the 4-kb space which can be inserted in the endogenous gene. We believe that HRE/CMV can be valuable in future gene therapy.

To better investigate the effect of HRE-regulated PR39 on IHD, AMI was modeled. Since miniature pigs have a coronary artery similar to that of humans with less collateral circulation (55,58), it is used for modeling. We constructed AMI models by introducing a balloon to block the coronary artery and verified the model by monitoring ECG and measuring troponin I and creatine kinase isoenzymes at different stages.

Delayed perfusion MR images at different stages of AMI models significantly distinguish normal and infracted myocardium. The infarcted area was displayed clearly. Significant differences in changes of myocardium over time post AMI were noted between the experimental and control group. The area of infarction in the control group changed slightly over time (no inflammatory edema period was taken into account). In the experimental group, the area of infarction decreased over time and the difference was statistically significant, as measured by the EFilm software. The left ventricular ejection fraction post AMI was also significantly different between the two groups.

Myocardial cells of the AMI miniature pigs receiving recombinant viruses and physical saline were analyzed using immunohistochemistry and the results showed that rAAV secreting PR39 could significantly increase the level of HIF-1α protein in hypoxic myocardial cells, thereby upregulating the expression of VEGF and promoting angiogenesis.

These animal experiments utilized only imaging data to show the ability of the recombinant virus to reduce the infarcted myocardial area and increase the HIF-1α protein in hypoxic myocardial cells. In this study, PR39 was administered immediately after AMI modeling and only pathological and immunohistochemical examinations were performed. However, in clinical practice, most AMI patients are not treated in a timely manner but are treated several hours post onset. Therefore, quantitative studies of the effects of the recombinant virus on IHD gene therapy should be performed to investigate the efficacy of the recombinant virus in this study following longer periods of AMI.
